# Dependence receptors: new targets for cancer therapy

**DOI:** 10.15252/emmm.202114495

**Published:** 2021-09-20

**Authors:** Morgan Brisset, Mélodie Grandin, Agnès Bernet, Patrick Mehlen, Frédéric Hollande

**Affiliations:** ^1^ Department of Clinical Pathology, Victorian Comprehensive Cancer Centre The University of Melbourne Melbourne Vic. Australia; ^2^ University of Melbourne Centre for Cancer Research Victorian Comprehensive Cancer Centre Melbourne Vic. Australia; ^3^ Apoptosis, Cancer and Development Laboratory Centre de Recherche en Cancérologie de Lyon, INSERM U1052‐CNRS UMR5286 Centre Léon Bérard Université de Lyon Lyon France

**Keywords:** apoptosis, cancer hallmarks, treatment resistance, tumor progression, Autophagy & Cell Death, Cancer, Signal Transduction

## Abstract

Dependence receptors are known to promote survival and positive signaling such as proliferation, migration, and differentiation when activated, but to actively trigger apoptosis when unbound to their ligand. Their abnormal regulation was shown to be an important feature of tumorigenesis, allowing cancer cells to escape apoptosis triggered by these receptors while promoting in parallel major aspects of tumorigenesis such as proliferation, angiogenesis, invasiveness, and chemoresistance. This involvement in multiple cancer hallmarks has raised interest in dependence receptors as targets for cancer therapy. Although additional studies remain necessary to fully understand the complexity of signaling pathways activated by these receptors and to target them efficiently, it is now clear that dependence receptors represent very exciting targets for future cancer treatment. This manuscript reviews current knowledge on the contribution of dependence receptors to cancer and highlights the potential for therapies that activate pro‐apoptotic functions of these proteins.

## Graphical abstract

Upon activation, dependence receptors promote survival, proliferation and migration but when unbound to their ligand, they trigger apoptosis. Here, F. Hollande & colleagues review the current knowledge on their contribution to cancer and discuss the therapeutic potential of targeting these receptors.

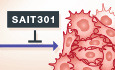

**Glossary**
Angiogenesis: Formation of new blood vessels.
Apoptosis: Programed cell death.
Autocrine/paracrine ligand: Molecule secreted by a given cell and acting on the cell itself (autocrine) or on neighboring cells (paracrine).
Chemoattractant: Substance capable of attracting motile cells that respond to it.
Colorectal carcinoma: Bowel cancer.
Dimerization, oligomerization: Process of joining two (di‐) or more (oligo‐) individual subunits of a protein, individually named monomers. Joined subunits can be the same (homodimerization) or different (heterodimerization).
Extracellular matrix: Network of proteins and other molecules that surround and support cells and tissues in the body.
Feedback loop: Control mechanism whereby an agent (e.g., protein) acts to regulate the process that controls its own production or activity.
Immune checkpoint inhibitors: Molecules used to block a type of inhibitory interaction between immune cells and their target, often used to restore adequate immune response against tumor cells.
Ligand pleiotropy: Ability of secreted molecules to modulate several, sometimes unrelated effects, e.g., by binding to different receptor types.
Metastatic spread: Dissemination of cancer cells from their organ of origin to distant organs throughout the body.
Mitochondria: Specific structure (organelle) found within most cells and where biochemical processes that enable cell respiration and energy production occur.
Morphogenesis: In the context of an animal or human, generation of tissue organization and shape.
Neutralizing antibody: Antibody molecule with the ability to block the function of its target protein.
Phenotypic switch: Rapid change in cell behavior/characteristics and/or structure.
Pluripotency markers: Genes and/or proteins that demonstrate characteristically high expression in stem cells that have the potential to give rise to all cells of the adult body.
Tyrosine kinase: Protein with the ability to drive the transfer of phosphate molecules onto tyrosine amino acids located within itself and/or other target proteins, leading to modifications in their structure/location/behavior.

## The dependence receptor paradigm

**Figure 1:**
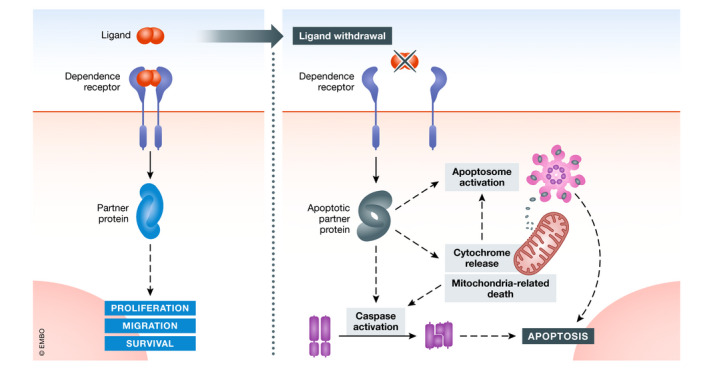
Dependence receptor paradigm Schematic representation of DR triggering: positive signaling in the presence (Top) and negative signaling in the absence of ligand (Bottom) (Created with BioRender.com).

For a long time, the accepted paradigm regarding transmembrane receptors was solely that their activation by specific ligands could lead to the initiation of signaling cascade transduction, resulting in various outcomes such as cell proliferation, motility, or cell survival. According to this model, the absence of ligand‐receptor binding was simply thought to result in the absence of intracellular signal transduction. However, the dependence receptor paradigm, first reported by Rabizadeh *et al* (1993) with a study of the p75NTR receptor (Rabizadeh *et al*, 1993), has changed our way of understanding molecular signaling downstream from transmembrane receptors. Indeed, dependence receptors (DRs) share a common feature: They actively trigger opposite outcomes depending on the availability of their ligand. These receptors, such as classical receptors, activate positive signaling in the presence of their ligands, but they also actively trigger apoptosis in their absence (Fig 1).

To date, more than twenty DRs have been identified within structurally divergent receptor families. Simply put, their shared property is not structural but a paradoxical pro‐survival/pro‐cell death function. Several DRs belong to the class of tyrosine kinase receptors. Indeed, receptors such as c‐MET (Tulasne *et al*, 2004), ALK (Mourali *et al*, 2006), TrkA and TrkC (Nikoletopoulou *et al*, 2010), RET (Bordeaux *et al*, 2000), EphA4 (Furne *et al*, 2009), EphB3 (Tsenkina *et al*, 2015), or even insulin receptor and insulin‐like growth factor 1 receptor (Boucher *et al*, 2010) and more recently c‐KIT (Wang *et al*, 2018) have been characterized as DRs. Receptors involved in major developmental signaling pathways such as hedgehog (SHH receptors patched‐1) (Thibert *et al*, 2003) and CDON (cell adhesion associated, oncogene regulated)(Delloye‐Bourgeois *et al*, 2013)) or NOTCH (NOTCH3 receptor) are also part of this class of receptors. The p75NTR neurotrophin receptor (Rabizadeh *et al*, 1993), the Dickkopf‐related protein 1 (DKK1) receptor Kremen1 (Causeret *et al*, 2016), and the GPCR receptor latrophilin (Jackson *et al*, 2016) also exhibit this feature. DRs also include transmembrane receptors binding netrin‐1 (a laminin related protein involved in axon guidance), such as deleted in colorectal carcinoma (DCC), uncoordinated 5 homologs (UNC5H (A‐D)) and neogenin (Llambi *et al*, 2001; Arakawa, 2004; Matsunaga *et al*, 2004; Mille *et al*, 2009), although the latter may not display any real dependence toward Netrin‐1 but rather toward the repulsive guidance molecule (RGM). Finally, even some integrin subtypes such as α5β3 and α5β1 (Stupack *et al*, 2001; Stupack, 2005) have been shown to actively trigger apoptosis when they are unbound.

Despite their divergent structure, most DRs regulate a small number of related biological processes: neuronal development and embryonic morphogenesis. Moreover, in many cases, their ligand‐dependent activation results in the stimulation of common signaling pathways, including PI3K/AKT and MAPK/ERK. These similarities may suggest that DRs act in a coordinated manner to modulate physiological functions such as neuronal development or embryonic vasculogenesis.

### The dependence function of DRs: ligand‐independent activation of apoptotic signals

Conversely, multiple DRs also show similarities in the way they trigger apoptosis. For example, in most cases, DR monomerization in the absence of ligand appears to initiate the apoptotic cascade through activation of various pro‐apoptotic protein partners (Negulescu & Mehlen, 2018). However, there are some exceptions to this paradigm. For instance, it was recently reported that withdrawal of DKK1 triggers the oligomerization of Kremen1, rather than its monomerization (Sumia *et al*, 2019).

The next step of ligand‐independent DR activation of apoptotic pathways frequently involves cleavage of an intracellular receptor moiety, a process that remains only partially understood. This initial step of DR activation appears heavily dependent on pro‐apoptotic proteases, including but not restricted to caspases. In some cases, “non‐apoptotic” proteases such as calpains, serine proteases, or lysosomal proteases have been shown to induce DR cleavage, with cleavage products sometimes initiating caspase‐dependent downstream signaling (Huai *et al*, 2010). Another mechanism appears to rely on the previously reported finding that non‐apoptotic cells have a baseline level of caspase activation, which plays a role in regulating differentiation during tissue development (Svandova *et al*, 2018). Such level of caspase activity is insufficient to trigger apoptosis but may be sufficient to initiate DR cleavage upon ligand withdrawal (Negulescu & Mehlen, 2018), suggesting that changes in DR oligomerization and/or conformation may be sufficient for them to reach a caspase‐sensitivity threshold. Accordingly, DR amino acid sequences often contain caspase‐like cleavage sites juxtaposed to an aspartate (Bredesen *et al*, 2005), a well‐known feature of caspase cleavage sites (Bratton & Cohen, 2001). Intracellular region within DR frequently exhibits unique a caspase‐like cleavage site, as seen in NOTCH3, DCC, neogenin, PTCH‐1, ALK, EphA4, EphB3, c‐KIT, CDON, as well as in the UNC5H receptor family, while multiple cleavage sites have been reported for other DRs such as RET, MET, and TrkC (Negulescu & Mehlen, 2018; Wang *et al*, 2018). In contrast, integrin isoforms previously identified as DRs do not exhibit any recognized cleavage site, even though β‐integrins induce apoptosis through recruitment and downstream activation of caspase‐8 (Stupack *et al*, 2001). Similarly, no cleavage sites were identified for p75NTR, latrophilin, IR, IGF‐1R, suggesting that early ligand‐independent signaling downstream from these receptors may not involve their cleavage.

Interestingly, caspases responsible for DR cleavages involve both “initiator” (caspases 2, 8, 9, 10, and 12) and “effector” caspases (caspases 3, 6, and 7). For instance, upon withdrawal of their respective ligand, the intracellular regions of UNC5B or c‐KIT are cleaved by caspase‐3, UNC5D can be cleaved by caspase‐2 or 3, while EphB3 can be cleaved by caspase‐8 or 9 (Abbaspour Babaei *et al*, 2016; Tsenkina *et al*, 2020; Zhu *et al*, 2021). Moreover, studies reveal that DCC can interact with caspase‐3 or caspase‐9 depending on its conformation, suggesting implication of one of these caspases in DCC cleavage (Forcet *et al*, 2001).

DR cleavage by caspases leads to their conformational modification, most frequently resulting in the exposure of a dependence domain of these receptors that remains anchored to the extracellular membrane. In contrast, the dependence domain of a few DRs (c‐MET, RET, TrkC, c‐Kit, and UNC5H receptors) is released in the cytoplasm (Bordeaux *et al*, 2000; Tulasne *et al*, 2004; Wang *et al*, 2018; Zhu *et al*, 2021). Exposure or release of dependence domains leads to the recruitment and activation of apoptotic partners, most frequently caspases. DRs such as c‐KIT, DCC, RET, TrkC, CDON, Notch3, PlexinD1, or PTCH‐1 trigger apoptosis through activation of caspases 3 and 9, whereas p75 NTR acts through caspase 2 activation.

Thus, DRs appear to play a major role in the amplification of caspase signaling. Indeed, on the one hand some level of pre‐existing caspase activation appears required to initiate pro‐apoptotic signaling from several DRs following withdrawal of their ligand, through cleavage of these receptors. In turn, most of these receptors also activate caspases and thereby contribute to the emergence of pro‐apoptotic signaling “waves”. DRs act as an intermediate between “initiator” and “effector” caspases, and their ligand‐independent function could also synergize with other effectors to amplify pro‐apoptotic signals. An example of such positive feedback loop involves p53 which, beyond its direct role in promoting apoptosis, also mediates upregulation of UNC5B and thereby leads to enhanced sensitivity to Netrin‐1 deprivation [34].

It is also noteworthy that apoptotic cascades triggered by some DRs do not always involve downstream recruitment of caspases but rather involve formation of pro‐apoptotic complexes with various other protein partners. The nature of recruited partners may not correlate with receptor structure, as exemplified by the UNC5H receptor family. Indeed, contrary to what structural homology among members of this family may suggest, these receptors trigger different apoptotic complexes involving distinct apoptotic partners. For example, upon Netrin‐1 withdrawal, monomerization of UNC5A activates the c‐Jun apoptotic pathway through its interaction with the neurotrophin receptor‐interacting MAGE homolog NRAGE (Williams *et al*, 2003). In contrast, UNC5B monomerization leads to PP2A (protein phosphatase 2A) recruitment and to the subsequent dephosphorylation and activation of DAPK1 (death‐associated protein kinase 1) (Llambi *et al*, 2005), a mechanism inhibited by CIP2A (cellular inhibitor of PP2A) in the presence of Netrin‐1. UNC5D is cleaved by caspases 2 or 3, and the resulting intracellular fragment interacts with E2F1 (E2F transcription factor 1) into the nucleus to activate transcription of pro‐apoptotic genes (Zhu *et al*, 2013). Finally, although pro‐apoptotic mechanisms downstream from UNC5C remain less characterized, UNC5C intracellular cleavage by γ‐secretase upon Netrin‐1 deficiency has been observed in Alzheimer disease and may also occur in other models (Chen *et al*, 2021).

In addition, pro‐apoptotic cascades triggered by several other DRs involve direct modulation of mitochondrial function. For example, NT‐3 withdrawal induces an internal cleavage of TrkC, followed by translocation of the cleaved intracellular portion into mitochondrial membrane via the protein Cobra 1 (cofactor of BRAC1). In turn, this leads to permeabilization of the mitochondria, release of cytochrome C and eventually to cell death via apoptosome activation (Ichim *et al*, 2013). The mechanism for c‐MET‐induced cell death also involves mitochondrial membrane permeabilization. Upon HGF withdrawal, MET is cleaved to yield a 40 kD intracellular fragment called p40MET (Foveau *et al*, 2007). This fragment localizes to mitochondria‐associated membrane (MAM) regions of the endoplasmic reticulum and triggers a calcium transfer from the endoplasmic reticulum toward the mitochondria. Massive Ca^2+^ entry into the mitochondria leads to mPTP (mitochondrial permeability transition pore) opening and to the release of mitochondrial components that trigger cell apoptosis (Duplaquet *et al*, 2020). PlexinD1 interacts with the nuclear receptor NR4A1 (nuclear receptor subfamily 4 group A member 1) to induce cell death through cytochrome‐c release and caspase‐9 activation (Luchino *et al*, 2013). Finally, ALK cleavage by caspase‐3 or another caspase facilitates apoptosis, possibly by regulating the activity of BCL‐2 family members (Mourali *et al*, 2006).

Recent studies into mechanisms that underpin c‐MET function in the absence of ligand have unraveled unexpected complexities in its regulation of cell death. First, multiple cytoplasmic cleavage products from c‐MET have been described (Fernandes *et al*, 2019) and a 10 amino acid small c‐terminal cleavage product was found to inhibit apoptosis by blocking active site of caspase‐3 in hepatocytes (Ma *et al*, 2014). Second, the implication of c‐MET in necrosis, a caspase‐independent cellular death, has also been reported, with calcium stress leading to cleavage of c‐MET by both ADAM‐family membrane metalloproteases and calpains to generate a fragment similar to p40MET but unable to trigger apoptosis (Fernandes *et al*, 2019). Whether similar alternative pathways exist for other DRs to control cell death remains unanswered at this point.

Linked to their role in the regulation of apoptosis, a function for DRs in tissue homeostasis has been identified, with DR ligands being produced by cells contributing to the stem cell niche. In this context, DR signaling contributes to promoting stem cell maintenance and tissue growth when their ligand is present, but negatively regulates cell numbers through apoptotic signaling when ligand expression drops. This process can control tissue/organ size. For instance, c‐MET regulates neural stem cell compartment expansion and self‐renewal (Nicoleau *et al*, 2009), whereas Notch3 and its ligand Jagged‐1 control differentiation of progenitors in the upper airways (Mori *et al*, 2015).

This ability of DRs to switch from positive to negative signaling is highly relevant for their function in morphogenesis, as the ability to induce cell death in the absence of ligand could be a way to foster normal organ development. However, this property of DRs can also have major pathological implications, especially in cancer. Indeed, the continuing proliferation of cancer cells may be limited by DRs, as an increasing number of cells in a growing tumor will compete for available ligand.

**Figure 2:**
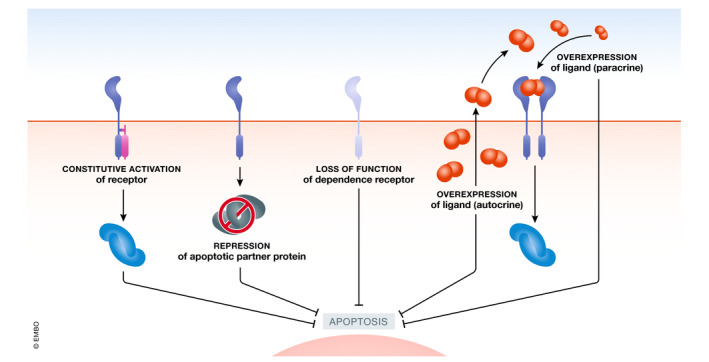
Mechanisms of dependence receptor‐triggered apoptosis escape in cancer Schematic representation of ways to escape DR‐triggered apoptosis: Constitutive activation of the receptor; repression of the DR apoptotic protein partner; DR loss of function; and autocrine or paracrine ligand overexpression) (Created with BioRender.com).

Collectively, these observations indicate that anomalies in the apoptotic pathways linked to DRs may confer a survival advantage to tumor cells. Accordingly, it is therefore not surprising that multiple DR pathway alterations have been reported in cancer (Fig 2), and alterations of DR signaling have been found to contribute to multiple steps in the tumorigenic process, as detailed below.

## Dependence receptors and tumorigenesis

The negative (or apoptotic) pathway downstream from various DRs is frequently altered in cancer, thus preventing apoptosis and favoring tumor growth. For example, genes that encode DRs such as DCC, neogenin, UNC5B, UNC5C, or TrkC are repressed in multiple cancer types (Bernet *et al*, 2007; Luo *et al*, 2013; Forrest *et al*, 2016; Kong *et al*, 2016; Xing *et al*, 2021), while cancer cells can also escape DR‐mediated apoptosis through autocrine overexpression of ligands including DKK1 (Huang *et al*, 2014), Netrin‐1 (Kefeli *et al*, 2017), NT‐3 (Louie *et al*, 2013), Sema3E (Yong *et al*, 2016), or HGF (Baykal *et al*, 2003). Alternatively, cancer cells can also stimulate the secretion of dependence ligands by cancer‐associated fibroblasts (CAFs), ensuring that pro‐apoptotic signaling by corresponding DRs is restrained (Sung *et al*, 2019).

**Figure 3:**
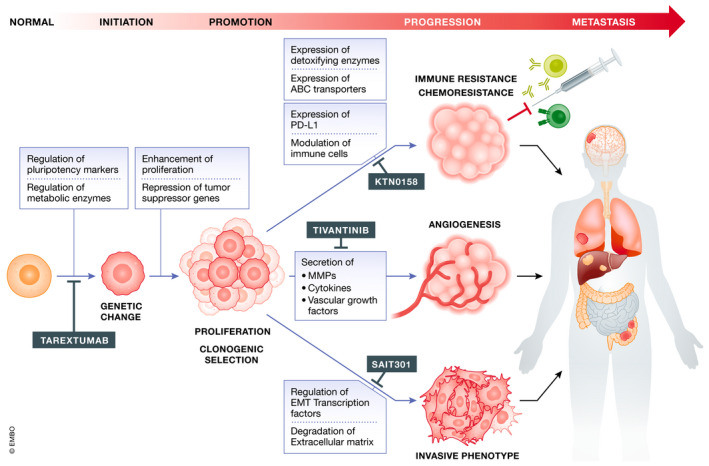
Involvement of dependence receptors in tumorigenesis and examples of drugs targeting them Impact of Dependence Receptors on different stages of tumorigenesis. Blue boxes highlight DR effects, while red boxes highlight examples of inhibitors that target them (Created with BioRender.com).

In addition, overexpression of DRs such as PlexinD1 (Blanc *et al*, 2011), NOTCH3 (Park *et al*, 2006), c‐KIT (Jones *et al*, 2007), c‐MET (Baykal *et al*, 2003; Zhang *et al*, 2017), PTCH‐1 (Kang *et al*, 2013), or TrkC (Jin *et al*, 2007) has been reported in several cancer types, in contexts where their ligands are also expressed. Indeed, joint overexpression of DRs and their ligand often promotes aggressive tumor progression, as was, for example, shown for HGF and c‐MET in NSCLC (Navab *et al*, 2009) or gastric cancer (Toiyama *et al*, 2012), suggestive of a positive selection for ligand‐induced rather than ligand‐independent signaling. Altogether, these observations suggest that silencing of the ligand‐independent pro‐apoptotic DR signaling combines with “positive” ligand‐driven DR signaling to confer a selective advantage to cancer cells, resulting in the promotion of multiple stages of tumorigenesis (see Fig 3 and Table 1).

**Table 1:** Examples reflecting the implications of DR in tumorigenesis.

	Ligand/Dependence receptor implicated	Effect on cancer cells	Cancer type	References
Initiation (Stemness)	c‐MET	Sox2, c‐Myc, Nanog, Klf4, Oct4 expression ALDH expression	Glioblastomas	Li, Y., *et al* Proceedings of the National Academy of Sciences, 2011b. 108(24): p. 9951–9956
		Sox2, Nanog, Oct4 expression, hexokinase‐2 expression	Pancreatic cancer	Yan, B., *et al* Experimental Cell Research, 2018. 371(1): p. 63–71
	Netrin‐1/Unc5B	CAFs secretion of pro‐stemness interleukins	Colon cancer and lung cancer	Sung, P.J., *et al* Cancer Res, 2019. 79(14): p. 3651–3661
	Notch3	Oct4, Nanog, Klf4, Rex1, Sall4, Rif1, NAC1 expression	Ovarian cancer	Park, J.T., *et al* The American journal of pathology, 2010. 177(3): p. 1087–1094
		β‐catenin inhibition, Nanog expression	Hepatocellular carcinomas	Zhang, Q., *et al* Oncotarget, 2015. 6(6): p. 3669–3679
	c‐KIT	Lgr5, CD44, OLFM4, BMI‐1, β‐catenin expression	Colorectal cancer	Siemens, H., *et al* Oncotarget, 2013. 4(9)
Promotion (Proliferation)	Notch3	Cell cycle progression, Hif‐2α regulation	Renal cancer	Han, Q., *et al* Oncology letters, 2020. 20(6): p. 379–379
	P75NTR	P53 repression	Skin, breast, bone and brain cancers	Zhou, X., *et al* eLife, 2016. 5: p. e15099.
Progression (Angiogenesis)	c‐MET	PI3K pathway activation	Breast cancer	Garcia, S., *et al* Int J Oncol, 2007. 31(1): p. 49–58
	TrkA	MMP2, NOS, VEGF expression	Breast cancer	Romon, R., *et al* Molecular cancer, 2010. 9: p. 157–157
	ALK	VEGF, VEGFR2, MMP2, MMP9 expression	Neuroblastoma	Di Paolo, D., *et al* Molecular Therapy, 2011. 19(12): p. 2201–2212
	c‐KIT	IL‐3, CXCL12, VEGFA production by mesenchymal cells	Breast cancer	Li, W., H. Xu, and C. Qian, BioMed Research International, 2017. 2017: p. 7407168
Progression (EMT)	c‐MET	MMP1, MMP2, MMP9 expression E‐cadherin repression	Colon cancer	Kermorgant, S., *et al* Carcinogenesis, 2001. 22(7): p. 1035–1042
		STAT3/Zeb1 activation	Gastric cancer	Cheng, Y., *et al* Translational Oncology, 2018. 11(2): p. 487–497
	Unc5B/Neogenin	MAPK pathway activation	Medulloblastoma	Akino, T., *et al* Cancer Research, 2014. 74(14): p. 3716–3726
	Neogenin	Zeb1 upregulation, PI3K/Akt activation	Gastric cancer	Qu, H., H. Sun, and X. Wang, Cellular Physiology and Biochemistry, 2018. 48(4): p. 1457–1467
	P75NTR	MMP12 and cadherin‐11 upregulation	Glioblastomas	Berghoff, J., *et al* Molecular and Cellular Neuroscience, 2015. 69: p. 41–53
Chemo‐ and immune resistance	c‐MET	ALDH expression	Glioblastomas	Li, Y., *et al* Proceedings of the National Academy of Sciences, 2011b. 108(24): p. 9951–9956
	P75NTR	Mitotical quiescence	Esophageal squamous cell carcinoma	Yamaguchi, T., *et al* Int J Oncol, 2016. 48(5): p. 1943–1954
	Notch3	ATP‐binding cassette expression	Ovarian cancer	Park, J.T., *et al* The American journal of pathology, 2010. 177(3): p. 1087–1094
		PD‐L1 expression	Breast cancer	Fatmah, A., *et al* OncoImmunology, 9:1, https://doi.org/10.1080/2162402X.2020.1729299

### Negative selection pressure on pro‐apoptotic DR signaling pathways in cancer

In view of the pro‐apoptotic activity initiated by DRs in the absence of ligand, it is somewhat unsurprising that this function, and by extension the occurrence of high receptor‐to‐ligand ratios that would foster the activation of ligand‐independent signaling, can be negatively selected in human cancer. Indeed, prior studies have identified multiple mechanisms of DR gene genetic invalidation (as in the case of DCC in colorectal cancer) (Fearon & Vogelstein, 1990), epigenetic downregulation (e.g., for UNC5H family receptors) (Thiébault *et al*, 2003), or sporadic mutations leading to selective loss‐of‐pro‐apoptotic function (e.g., for TrkC in colorectal cancer) (Ichim *et al*, 2013). Other mechanisms of DR pro‐apoptotic pathway abrogation include the constitutive activation of positive signaling by the ALK receptor through gene fusion with EML4 (Wei *et al*, 2018) or the epigenetic alteration of pro‐apoptotic signaling cascade proteins such as DAPK1 (apoptotic partner of UNC5B) (Grandin *et al*, 2016).

A causal relationship between DR inactivation and tumor progression was, for example, demonstrated in the case of UNC5C, which is downregulated via LOH and epigenetic silencing in colorectal cancer, whereby *in vivo* UNC5C inactivation increased tumor progression and decreased apoptosis in an APC1638N spontaneous intestinal tumors mouse model. Negative pressure on pro‐apoptotic function can also result from constitutive ligand overexpression in the tumor environment (e.g., Netrin‐1) (Fitamant *et al*, 2008), which enables consistent ligand/receptor engagement.

### Role of DRs in tumor initiation

Activation of these receptors may provide a facilitating environment for the initiation of tumorigenesis. Cancer stem cells (CSCs), a subpopulation of cancer cells that exhibit long‐term self‐renewal ability, are essential contributors to tumor initiation and maintenance of tumor growth (Prasetyanti & Medema, 2017), and a plethora of studies demonstrates the importance of DR activation in promoting their stem‐like cell behavior. For instance, c‐MET plays an important role in maintaining stemness in pancreatic cancer cells (Li *et al*, 2011a). Its activation results in enhanced expression of pluripotency markers NANOG, OCT4, and SOX2, through activation of YAP and potentiation of the Warburg effect (Yan *et al*, 2018). In fact, the activation of multiple DRs has been shown to regulate the expression of pluripotency markers. For example, in ovarian cancer, the activation of c‐KIT enhances tumor initiation *in vivo* through activation of PI3K/AKT and Wnt /β‐catenin/TCR pathways. The crosstalk between cancer cells and cells from their microenvironment, such as cancer‐associated fibroblasts (CAFs), also promotes plasticity and self‐renewal through DR activation. For instance, in colon and lung cancers, cancer‐associated fibroblasts (CAFs) secrete Netrin‐1 and take part in a two‐way signaling process with cancer cells, which involve the secretion of stemness‐promoting cytokines such as interleukin‐6 (IL‐6), resulting in an increased stemness phenotype of cancer cells (Sung *et al*, 2019). These studies and multiple others (Huang *et al*, 2009; Park *et al*, 2010; Kimura *et al*, 2011; Li *et al*, 2011b; Xu *et al*, 2020) highlight the involvement of DRs in tumor initiation through the regulation of pluripotency markers, metabolic enzymes, and other important features of CSCs.

### DR contribution to tumor progression

Tumor promotion involves a phase of clonal expansion. During this step, tumor cells undergo sequential genetic modifications that drive malignant transformation and enable escape from tumor suppressors. Therefore, during this step of tumorigenesis, a prevalent aspect is proliferation of tumor cells, and DR activation has major implications in the promotion of this hallmark. Thus, NOTCH3 was shown to promote the proliferation of renal cancer cells through regulation of cell cycle progression and of hypoxia‐inducible transcription factor 2‐alpha (HIF‐2α) expression (Han *et al*, 2020). Another example is p75NTR activation, which enhances the clonogenic capabilities of cancer cells by inactivating p53 through activation of MDM2‐mediated ubiquitylation (Zhou *et al*, 2016). These are representative illustrations of the combined DR involvement in promoting proliferation and escaping tumor suppressor gene‐driven apoptosis, supporting transformation of a preneoplastic lesion into a malignant one and subsequently promoting tumorigenesis.

Angiogenesis constitutes another major aspect of tumor progression. The formation and remodeling of new blood vessels and capillaries are essential to compensate the tumor core hypoxia that often accompanies uncontrolled proliferation of cancer cells. Pathways triggered by DRs are critical for different aspects of angiogenesis modulation, including the secretion of vasculogenesis‐promoting cytokines, of protein involved in extracellular matrix remodeling such as matrix metalloproteinases (MMPs), and in the expression of vascular growth factors such as VEGF and its receptors. These factors can be produced by cancer cells themselves, as exemplified by the secretion of several MMPs and of VEGF in neuroblastoma cells expressing ALK (Di Paolo *et al*, 2011). They can also be secreted by endothelial cells surrounding the tumor, as demonstrated by the increased levels of MMP2, NOS, and VEGF in endothelial cells following activation of signaling pathways such as PI3K and ERK downstream of NGF‐activated TrkA, leading to enhanced invasion, cord formation, and monolayer permeability in a breast cancer model (Romon *et al*, 2010). Interestingly, Netrin‐1 can also function as a survival factor for endothelial cells by blocking UNC5B apoptotic activity. Thus, Netrin‐1 not only promotes tumor progression directly but may also enhances the development and survival of new vessels within the tumor (Mehlen & Guenebeaud, 2010) Moreover, Netrin‐1 also plays a role in vasculogenic mimicry (VM) (Zhang *et al*, 2018) (Zuazo‐Gaztelu & Casanovas, 2018). Importantly, VM and neo‐angiogenesis promote metastatic spread, as newly formed vessels provide a route by which tumor cells can leave the primary tumor site and invade other sites in the body (Folkman, 2002; Takeda *et al*, 2002).

### Involvement of DRs in metastasis development

The development of distant metastases is a key feature of aggressive tumor progression and has important implications for patient outcome. Multiple studies report the role of dual signaling from DRs in regulating the metastatic spread of tumor cells, with DR‐related cell death hindering tumor cell migration to and colonization of sites where ligands are absent, and overexpression of DR ligands conversely providing a selective advantage during metastasis development. It follows that escaping DR‐induced apoptotic death is an important step to foster metastatic dissemination of tumor cells as well as their survival at metastatic sites. For instance, the overexpression of Netrin‐1 was reported to confer a survival advantage to metastatic cells, acting through the inhibition of DCC and UNC5H receptors apoptotic activities. This likely explains why Netrin‐1 is overexpressed in metastatic breast cancer whereas its expression in non‐metastatic tumors is lower (Fitamant *et al*, 2008). Similarly, co‐expression of c‐MET and its ligand HGF was shown to predict peritoneal dissemination in gastric cancer (Toiyama *et al*, 2012).

Besides the previously discussed role of angiogenesis as a facilitator of metastatic dissemination, tumor cells themselves often undergo a phenotypic switch that enables them to acquire invading capacities. Indeed, epithelial‐to‐mesenchymal transition (EMT), a well‐described phenotypic switch involved in metastasis development, is one of the fields where DR regulation has been the most studied across various cancer types. DR‐driven activation of PI3K and ERK pathways activates critical EMT transcription factors such as ZEB, TWIST, and SNAIL. For instance, the activation of TrkA in head and neck carcinomas activates STAT3, which in turn increases the expression of Twist1, Snail1, and Snail2, thereafter leading to decreased E‐cadherin expression and increased levels of vimentin and N‐cadherin (Lin *et al*, 2020). These transmembrane and cytoskeleton proteins are major regulators of cellular adherence and morphology, and their modulation is an essential step in the EMT process. Moreover, DRs also contribute to the metastasis process by modulating extracellular matrix composition through increased secretion of MMPs, as mentioned earlier, thus facilitating migration of cells. An additional example is the activation of c‐MET receptors by HGF, which results in enhanced production by cancer cells of MMP1, MMP2, and MMP9, enzymes that can degrade extracellular matrix proteins. In addition to its effects on E‐cadherin and MMPs, it has been found that c‐MET regulates EMT in cancer cells through the activation of hBνR (human biliverdin reductase), an ERK nuclear transporter required for MAPK signaling (Lerner‐Marmarosh *et al*, 2008; Liu *et al*, 2017). This impact of c‐MET on EMT has a drastic impact on cancer cell invasiveness and metastatic abilities, and is reflected in the upregulation of both c‐MET and HGF in circulating tumor cells (CTCs), as shown, for example, in hepatocellular carcinoma (Ogunwobi *et al*, 2013). In addition, EMT has been linked to increased chemotherapeutic resistance of cancer cells through enhancing of cancer cell survival, cell fate transition, and upregulation of drug resistance‐related genes (Wang *et al*, 2016), suggesting that DRs may have a role in regulating this process.

### DRs and treatment response

Indeed, numerous studies show a link between the expression of DRs in cancer cells and resistance to different types of treatments. Correlations between expression of c‐MET and resistance to cisplatin and ionizing radiations in several types of cancer (De Bacco *et al*, 2011; Sun & Wang, 2011), or between NOTCH3 expression and enhanced cisplatin resistance (Park *et al*, 2010), are good illustrations of their implications. This role may directly result from their effect on cell cycle and on the expression of detoxifying enzymes such as aldehyde dehydrogenase (ALDH) and ATP‐binding cassette (ABC) transporters, transmembrane proteins that mediate drug export out of the cell (Park *et al*, 2010; Liu *et al*, 2018; Prager *et al*, 2019). Another reason underlying the correlation between DR expression and resistance to chemotherapies could be the positive impact of these receptors on angiogenesis, since high vascular density was shown to associate with a decreased response to chemotherapies (Mattern, 2001).

### A role for DRs in immune escape?

In addition, besides resistance to chemotherapies, DRs may also be implicated in the ability of tumor cells to escape immune surveillance in several cancer types. The correlation between NOTCH3 and PD‐L1 expression in breast cancer, and especially in breast cancer stem cells, is a good illustration of this. Indeed, PD‐L1 expression is modulated by NOTCH3 via the activation of mTOR signaling (Mansour *et al*, 2020). In addition, DRs have a significant impact on the migration and maturation of immune cells. For instance, neogenin has been described as an important regulator of neutrophil migration and an activator of inflammation in a lung injury model (Mirakaj *et al*, 2012). In the context of cancer, the activation of NGF‐TrkA signaling axis is an important step to produce IL‐10 in tumor‐associated macrophages (TAMs) (Ley *et al*, 2013). These examples among others illustrate the modulatory effect of DRs on immune cells and their involvement in tumor immune escape.

Thus, from tumor initiation to the promotion of metastasis development and the avoidance of immune cell regulation, DRs display multiple implications along multiple steps of tumorigenesis (Table 1), highlighting their potential as a therapeutic target in cancer.

## Dependence receptors and clinical treatments

### Targeting dependence receptor pathways: a promising avenue for cancer therapy

In view of their multi‐faceted role in the regulation of cancer hallmarks, DRs are emerging as very promising potential targets in oncology (Fig 3). Blocking interactions between these receptors and their ligands not only would trigger apoptotic death of cancer cells, but could also prevent metastatic spread, immune escape, and resistance to chemotherapy by inhibiting positive signaling from DRs responsible for EMT, stemness, angiogenesis, and immuno‐modulation. A lot of molecules targeting these receptors are being evaluated in clinical trials. Even though the dependence function has not directly been considered in most of these trials, results from some do highlight therapeutic potential. As discussed above, such molecules could have an impact on all step of tumorigenesis, from the tumor initiation to the metastatic dissemination.

We have seen that DRs can have an impact on stemness features in some cancer cells and that their activation supports the potential of tumor‐initiating cells. Preventing their activation could therefore have a significant impact on post‐treatment recurrence. For instance, Tarextumab, a Notch2/Notch3 monoclonal antibody, was shown to inhibit tumor growth and to decrease tumor‐initiating cell frequency in preclinical studies. Unfortunately, the phase II clinical trial evaluating the efficacy of Tarextumab on metastatic pancreatic cancer (NCT01647828) did not show any improvement in patient survival (Hu *et al*, 2019), but its efficacy is still being tested for other cancer types (NCT01277146). Thus, targeting DRs may not only diminish cancer stem cell plasticity but potentially inhibit interactions between CSC and niche cells, including CAFs, thereby minimizing cancer recurrence if used in combination with conventional chemotherapies.

Since DRs can modulate early steps of the tumorigenesis process, targeting them could also be used as an innovative early‐stage therapy, preventing key events that foster tumor growth such as angiogenesis. For instance, current clinical trials evaluate the combination of anti‐angiogenic drugs and c‐MET inhibitors to enhance anti‐angiogenic killing of cancer cells, as exemplified by the combination of pazopanib and ARQ 197, also called tivantinib, a highly selective c‐MET inhibitor, in renal cancer (NCT01468922). The same principle was followed in a clinical trial combining bevacizumab (Avastin), an anti‐angiogenic drug already used in several cancer types and INC280, a highly potent and selective c‐MET inhibitor (NCT02386826). Based on preclinical results demonstrating a synergistic effect between c‐MET and VEGFR inhibitors in mice (Harshman & Choueiri, 2013; Cascone *et al*, 2017), these phase 1 clinical trials show the potential benefits of targeting c‐MET, and more generally, DR‐associated angiogenesis. Indeed, INC280 has already been approved by the United States FDA (Food and Drug Administration) for the treatment of NSCLC (East Hanover).

The impact of therapies targeting DRs on angiogenesis, such as the combinations described above, could also decrease metastatic spread. Since most DRs also have a direct impact on invasion and migration of cancer cells, their targeting could potentiate this anti‐metastatic effect, for example, by preventing EMT. SAIT301, a humanized monoclonal antibody targeting the c‐MET extracellular domain alpha chain, provides a good illustration of this potential. It was shown to provide significant benefit in a phase I trial (NCT02296879) and to reduce invasion and migration of nasopharyngeal cancer cells (Bhatia *et al*, 2017). Migration and invasion of cancer cells were also inhibited by another monoclonal antibody targeting c‐MET, YYB‐001 (Kim *et al*, 2019). A phase I clinical trial with this antibody has just been completed for colorectal cancer (NCT04368507) (Kim *et al*, 2020), reflecting the potential of therapies targeting DRs for preventing metastatic spread.

As discussed above, DRs have also been shown to modulate interactions between tumor and immune cells, usually promoting the ability of tumor cells to evade the immune system. Consequently, targeting them could be an innovative way to decrease immune resistance of cancer cells and enhance the efficiency of immune checkpoint inhibitors, when used in combination. DR antagonists could also be used as single therapies against hematopoietic cancer cells. For example, the anti‐c‐KIT antibody KTN0158 (also called CDX‐0158) demonstrated a good efficacy against malignant mast cells in preclinical studies (London *et al*, 2017). This antibody is now undergoing phase I clinical trial (NCT02642016) and was shown to enhance the efficacy of immune checkpoint inhibitors such as CTLA‐4 or PD‐L1 antibodies (Garton *et al*, 2017). This synergistic effect between KTN0158 and checkpoint inhibitors has been attributed to SCF‐mediated control of mast cell development. Indeed, both SCF and KIT are expressed by several pro‐inflammatory cells such as mast cells, regulating their migration through activation of PI3K/AKT signaling. SCF acts as a chemoattractant to mast cells and is responsible for their infiltration in tumors. Mast cells activated by SCF participate in tumor immunosuppression, increasing regulatory T cells number and enhancing the suppression of other T and NK cells in tumors, having a major pro‐tumoral effect. This is the result of enhanced production of IL‐17 and other inflammatory cytokines by SCF‐stimulated mast cells. The presence of SCF‐activated mast cells results in higher levels of immunomodulatory cytokines and transcriptional regulators such as IL‐10, TGF‐β, and Foxp3, within tumors (Huang *et al*, 2008). This study elegantly exemplifies how using drugs targeting DRs could enhance immunotherapy efficacy.

Finally, while DRs represent promising therapeutic targets in multiple cancer types, analysis of their expression could also enable the development of prognostic and/or predictive biomarkers in oncology. For example, Netrin‐1 quantification in human fluids was shown to be a promising biomarker and a predictor of tumor recurrence in bladder, gastric, and lung cancers (Kefeli *et al*, 2012; Yıldırım *et al*, 2016; El‐Gamal *et al*, 2020), and to correlates with poor prognosis in patients with brain metastasis (Harter *et al*, 2014). Another example is the quantification of c‐MET expression, which provides predictive information on VEGFR tyrosine kinase inhibitor sensitivity in non‐small‐cell lung cancer (Cascone *et al*, 2017).

### Dependence receptors: how to target them efficiently

We have seen that DRs are promising targets for therapy in oncology, firstly because of their implication throughout all phases in tumorigenesis and secondly, because this kind of drug would not only prevent these cancer‐associated features but also lead to apoptosis in cancer cell harboring these receptors. We can then speculate that preventing these ligand‐DR associations could be an efficient way of treating tumors. Nevertheless, since the DR concept is relatively new, it is important to discuss how pathways regulated by these proteins can be targeted most efficiently.

First, the decision of whether to target the ligand or its receptor has important implications, with several factors to be considered. For example, ligand availability and abundance, as well as the affinity of a ligand for its receptor and its binding dynamics, are critical variables to take into consideration (Kim & Cochran, 2017). A key parameter to consider for this strategy is the possible ligand pleiotropy. For example, Netrin‐1 being a ligand for several DRs (DCC and UNC5H family receptors), targeting it offers an effective way to treat several types of cancers where one or several of its receptors is expressed. Conversely, SHH is a ligand for two DRs (PTCH‐1 and CDON), but also acts through binding to several other non‐DR receptors, suggesting that targeting only some of its receptors may allow a better control of subsequent biological effects. Moreover, the reported ability of some drugs targeting cell surface receptors to induce receptor downregulation, leading to a decreased presence of receptors at the membrane, should also be taken into consideration. Indeed, this phenomenon was reported to promote the therapeutic efficacy of a treatment with non‐DR receptors (Kim & Cochran, 2017) but could decrease efficacy of treatments targeting DRs, since a diminution of their expression at the membrane would lead to decreased apoptotic signaling. Therapeutic targeting of DRs therefore requires careful consideration of the number of different ligands and receptors (DRs and non‐DRs) involved, of the ligand bioavailability, and of the nature of downstream pathways implicated in a specific cancer type to achieve optimal efficiency.

Possible interactions between signaling pathways activated by different DRs may make their targeting more complex but could also reveal systems of cellular dependencies that are amenable to medical targeting. Ligand‐dependent DR signaling seems to mostly coordinate the activity of a small number of signaling pathways (ERK/MAPK, PI3K/AKT, Wnt, or TGF‐beta), implying that the net result of individual DR activation may vary depending on the cellular context of other similarly acting receptors. It is therefore plausible that the concomitant targeting of several of these DRs could have synergistic effects and may result in greater efficacy than the targeting of individual ones. Moreover, in view of the reported interaction and coordinated activation of similar signaling pathways by multiple DRs, targeting of several of these receptors concomitantly may preclude the emergence of resistance to drugs targeting single DRs. For example, targeting p75NTR could help bypass the observed resistance to tyrosine kinase inhibitors targeting TrkA or TrkC since it is involved in their regulation and seems to cooperate with them to induce several types of cellular responses (Barker & Shooter, 1994; Hantzopoulos *et al*, 1994; Jung *et al*, 2003).

Many existing drugs such as tyrosine kinase inhibitors in fact target DRs. Examples include the ALK and c‐MET inhibitor Crizotinib, the c‐KIT inhibitor Axitinib, as well as Entrectinib and Merestinib, which inhibit Trk receptors. However, these molecules act on DRs without preventing ligand binding. When considering the DR paradigm, it is therefore clear that these drugs inhibit positive signaling pathways triggered by these receptors, but that they are not expected to mimic the effects of ligand withdrawal and therefore to actively trigger apoptotic pathways within cancer cells. We argue that, in the case of DRs, alternative targeting approaches that result in abrogation of receptor activation, while inducing pro‐apoptotic signaling by the “ligand‐starved” receptor, should provide much more significant therapeutic benefit. Such is the case for monoclonal antibodies that inhibit survival pathways triggered by these receptors by preventing binding of their ligands. Such molecules are currently being developed and tested such as ficlatuzumab (NCT03422536), KTN0158 (London *et al*, 2017)(NCT02642016), or NP137 (Cassier *et al*, 2019b) which, respectively, inhibit the binding between HGF and c‐MET, SCF and c‐KIT, or Netrin‐1 and its receptors.

DRs responding to Netrin‐1, particularly DCC and UNC5B, have been studied extensively in cancer, with validation of efficacy of a therapeutic molecule that inhibits interactions between Netrin‐1 and its receptors in this context. For example, NP137, a humanized neutralizing monoclonal antibody against Netrin‐1, is currently undergoing phase Ib/II clinical trials for the treatment of gynecological cancers (NCT04652076) and phase 1b trials in advanced or metastatic solid tumors (NCT02977195)(Cassier *et al*, 2019a). Interestingly, despite the involvement of Netrin‐1 and its DRs in numerous pathways, no obvious toxicity has been observed in phase 1 trials (Cassier *et al*, 2019b).

Beyond their implication across multiple stages of tumorigenesis, DRs are also attractive targets because their mutation as well as the expression level of themselves and/or their ligands can predict sensitivity to their inhibitors. This opens the door for a precision oncology approach, whereby tumors that exhibit a high expression of DRs and their ligands may be eligible for treatment with relevant molecules targeting these receptor/ligand interactions. For instance, Larotrectinib (or LOXO‐101), a drug which targets all three Trk receptors, is now approved for treatment of several types of cancer after several clinical trials (NCT02122913, NCT02637687, and NCT02576431)(Hong *et al*, 2020). This molecule was developed for the treatment of cancers carrying NTRK gene fusions, regardless of cancer type (Roviello *et al*, 2020). The use of Larotrectinib suggests that specific targeting of some DRs driven by improved molecular tumor profiling should allow better and more effective treatments for patients.

While DRs represent interesting targets for cancer therapy, additional information must be gathered to document potential side effects for this type of treatment. Because these receptors are generally expressed at low levels in adults, side effects of molecules targeting them may be low, as already shown for the anti‐Netrin‐1 antibody mentioned above. Nevertheless, considering their implication in neuronal development, a special attention should be paid to possible neurological side effects during development of these new drugs. It is also likely that cancer cell plasticity will enable the appearance of some degree of resistance to this type of treatment. This could, for example, occur via inhibition of pro‐apoptotic signaling, for instance though repression of DAPK1 to disable UNC5B‐dependent apoptotic signaling in the absence of ligand (Grandin *et al*, 2016).

### DRs: latest advances and translational impact

Since the field of DR study is relatively new, recent and exciting discoveries in this area not only include additional mechanistic characterization of their role in various physio‐pathological settings, but also include the novel identification of additional receptors that exhibit a DR function. Notably, Wang *et al* recently observed that SCF withdrawal triggers apoptotic signaling through c‐KIT intracellular cleavage by caspases, resulting in apoptosome activation (Wang *et al*, 2018). Considering the broadly reported involvement of c‐KIT in several cancer types and in cancer stem cell regulation, this first identification of c‐KIT as a DR should have important repercussions for researchers aiming to develop molecules targeting this receptor (Stankov *et al*, 2014; Abbaspour Babaei *et al*, 2016), since DR characteristics of c‐KIT should be considered to fully exploit the therapeutic potential of this receptor.

Several elegant studies have also brought additional light on signaling mechanisms that drive the ligand‐independent activation of apoptotic pathways by DRs, with the description of mitochondria permeabilization triggered by p40MET (Duplaquet *et al*, 2020), of EphB3 cleavage by Dral/FHL‐2 and caspase‐8 or 9 (Tsenkina *et al*, 2020), and of UNC5C cleavage by active δ‐secretase all constituting important advances in the field. Findings from these studies not only further strengthen the concept of DR but also enable a better understanding of the DR signaling network, an essential step toward the rational design of new therapeutic molecules efficiently targeting these receptors. Along with these studies, the recent demonstration by Sumia *et al* that homo‐ and heterodimerization of Kremen1 is required to trigger apoptotic signaling in the absence of Dickkopf1 binding and that the Kremen2 paralogue receptor can act as an inhibitor of Kremen1‐induced cell death through direct competition with Kremen1, illustrates the underlying complexity of DR apoptotic signaling, and highlights an additional layer of DR regulation via direct interaction with neighboring transmembrane molecules.

Finally, an important recent development in the field has been the preclinical validation and clinical evaluation of NP137, a first‐in‐class monoclonal antibody targeting netrin‐1 and the first molecule designed to fully exploit the therapeutic potential of a DR (Cassier *et al*, 2019b). Following promising data from a phase I clinical trial of NP137 as single agent in patients with advanced refractory solid tumors, this antibody is currently assessed in additional trials, including in combination with the anti‐PD‐1 antibody pembrolizumab for patients with locally advanced/metastatic uterine tumors (NCT04652076), known to exhibit frequent Netrin‐1 overexpression. Beyond their direct impact on the survival of treated patients, results from these trials will represent an important step toward a better understanding of neutralizing monoclonal antibody as possible tools of choice to target DR, through the inhibition of ligand binding and the resulting activation of pro‐apoptotic DR signaling.

## Conclusion

We have sought here to define, explore, and analyze the concept of DRs and their implication in tumorigenesis. Indeed, through modulation of pathways such as ERK/MAPK or PI3K/AKT, activation of DRs influences tumorigenesis from initiation to metastasis. Altogether, studies involving DR pathways highlight the targeting of these receptors as an innovative and interesting approach for future cancer therapies. Indeed, preventing their activation could decrease the tumorigenic potential of cancer cells by limiting pro‐tumoral features such as proliferation, angiogenesis, and/or chemoresistance, as well as by preventing ligand binding to induce apoptotic cell death.

Nevertheless, before accessing the full therapeutic potential of these receptors, a better understanding of the pathways they transduce within cancer cells and of their interactions, direct or indirect, is needed. Moreover, an important amount of preclinical and clinical studies is required to define what is the best way to target these receptors for which tumor type and to further characterize potential side effects. These studies represent an essential step toward unraveling the real potential of dependence receptor targeting for the benefit of cancer patients.

**Pending issues**

- Determine whether the full scope of dependence receptor has yet been discovered.
- Characterize the extent and biological significance of cross‐signaling events between different dependence receptors.
- Establish how the initial activation of cleavage unfolds for most dependence receptors.
- Evaluate optimal targets for clinical translation (ligand vs receptor, single vs multitarget…)
- Optimize targeting of dependence receptors so as to concomitantly inhibit positive signaling and promote pro‐apoptotic signals.

## Conflict of interest

Patrick Mehlen and Agnès Bernet are shareholders of Netris Pharma, which develops a netrin‐1 antibody for clinical use. The other authors declare no conflict of interest.

## For more information

- F. Hollande lab: https://mdhs.unimelb.edu.au/centre‐for‐cancer‐research/our‐research/Tumour‐Heterogeneity‐in‐Metastatic‐Cancer
- P. Mehlen lab: https://www.crcl.fr/en/teri‐department/apoptose‐cancer‐et‐developpement/
